# *Rhodomyrtus tomentosa* fruit ameliorates LPS induced depression-like behaviors in mice by attenuating hippocampal neuroinflammation via inhibiting the TLR4/MyD88/MAPK/NF-κB/NLRP3 signaling pathway

**DOI:** 10.3389/fnut.2026.1806547

**Published:** 2026-04-23

**Authors:** Jingxi Zhang, Jinyuan Liang, Yan Xu, Jingyu Sun, Mengyue Zhang, Zhiyou Yang, Leigang Jin, Shaohong Chen, Yun-Tao Zhao, Chuanyin Hu

**Affiliations:** 1 Guangdong Province Engineering Laboratory for Marine Biological Products, Guangdong Provincial Key Laboratory of Aquatic Product Processing and Safety, Zhanjiang Municipal Key Laboratory of Marine Drugs and Nutrition for Brain Health, College of Food Science and Technology, Modern Biochemistry Experimental Center, Guangdong Ocean University, Zhanjiang, China; 2 State Key Laboratory of Pharmaceutical Biotechnology, Department of Medicine, The University of Hong Kong, Hong Kong, Hong Kong SAR, China; 3 School of Basic Medical Sciences, Guangdong Medical University, Zhanjiang, China

**Keywords:** BV2 cells, depression, neuroinflammation, *Rhodomyrtus tomentosa* fruit, TLR4/MyD88/MAPK/NF-κB/NLRP3 signaling pathway

## Abstract

**Introduction:**

*Rhodomyrtus tomentosa* fruit is a dietary food with various bioactivities. However, the comprehensive mechanisms underlying its antidepressant effects remain be fully understood.

**Methods:**

This study aimed to assess the effects of the ethanol extract from *R. tomentosa* (RTEE) on depression-like behaviors in lipopolysaccharide (LPS)-challenged mice, and to explore the molecular basis.

**Results:**

The results showed that RTEE significantly improved LPS induced depression-like behaviors in mice, as evidenced by increased preference for sucrose in the sucrose preference test, enhanced locomotor activity (elevated total distance, velocity, and center entries) in the open field test, and reduced immobility time in both the tail suspension and forced swim tests. Meanwhile, RTEE significantly increased NeuN^+^ cell numbers and enhanced PSD95 expression in the hippocampus, thus promoting neuronal survival and synaptic plasticity. Additionally, RTEE significantly suppressed microglial and astrocyte activation (indicated by reduced Iba-1^+^ and GFAP^+^ cell numbers), decreased the expression of hippocampal pro-inflammatory cytokines (TNF-α and IL-6) via inhibiting the TLR4/MyD88/MAPK/NF-κB/NLRP3 signaling pathway, thereby attenuating neuroinflammation. *In vitro* experiments using microglial BV2 cells showed that RTEE significantly rescued cell viability and reduced NO production via suppressing the NF-κB signaling pathway.

**Discussion:**

These findings suggested that RTEE improved LPS induced depression-like behaviors in mice by attenuating hippocampal neuroinflammation via inhibiting the TLR4/MyD88/MAPK/NF-κB/NLRP3 signaling pathway. Therefore, this study elucidates the underlying mechanisms through which *Rhodomyrtus tomentosa* fruit may serve as a novel nutritional intervention for depression.

## 1 Introduction

Depression is a prevalent psychiatric disorder that manifests as persistent affective dysregulation and significant social disengagement. Epidemiological studies reveal a rising prevalence of depression, peaking in the 60–64 age group with marked gender differences (1). Depression has been recognized as a primary etiological factor for both completed and attempted suicide, imposing a significant burden on global healthcare systems (2).

To date, the pathogenesis of depression remains incompletely understood. Multiple pathological mechanisms, including neuroinflammation, monoamine neurotransmitter deficiencies, hypothalamic-pituitary-adrenal (HPA) axis hyperactivity, and mitochondrial dysfunction contribute to the development and progression of depression. Conventional pharmacotherapy for depression primarily targets monoamine neurotransmitter systems, yet is hampered by a slow onset of action and substantial adverse effects (3). In contrast, exercise therapy demonstrates efficacy in improving depressive symptoms (4). However, its effectiveness is often hampered by patients’ social disengagement and self-isolation behaviors. In contrast, plant-based foods exhibit a favorable safety profile and enhanced acceptability, positioning it as a growing focus of research in depression management (5, 6).

In southern China, the Myrtaceous shrub *Rhodomyrtus tomentosa* (Ait.) Hassk produces edible berries traditionally consumed as a tonic, valued for its potential to alleviate diarrhea and enhance sexual function. Nevertheless, pharmacological studies have focused predominantly on the leaves, which have shown notable anti-inflammatory, anti-microbial, antitumor, antioxidant, and antidiabetic properties (7–9). By contrast, the fruit has been far less studied, which has demonstrated antioxidant, anti-inflammatory, gut protective, hepatoprotective and lipid-lowering effects (10–13). Despite established antidepressant effects in the chronic unpredictable mild stress model (14), its efficacy against acute lipopolysaccharide (LPS) induced depression-like behaviors has yet to be explored, necessitating further research to delineate its complete antidepressant profile.

To this end, the ethanol extract from *R. tomentosa* (RTEE) was prepared and evaluated for its impact on LPS induced depression-like behaviors in mice. Additionally, the molecular mechanisms behind the observed antidepressant effects were investigated.

## 2 Materials and methods

### 2.1 RTEE preparation

The RTEE used in this study was prepared from commercially available *R. tomentosa* fruit. The detailed extraction protocol and a comprehensive phytochemical characterization of this extract have been provided in our previous work (14). In brief, the powdered *R. tomentosa* fruit underwent extraction with 70% ethanol (1:10, w/v) using a combination of maceration (12 h) and ultrasonication (1 h). Following filtration and concentration, the resulting extract was freeze-dried to yield RTEE, which was stored at −20 °C.

### 2.2 Animal model and treatment

Male C57BL/6 mice (4 weeks old at arrival) bought from the Guangdong Medical Laboratory Animal Center (Guangzhou, China) were randomly assigned into six groups of eight: control, LPS, three LPS + RTEE groups receiving low (200 mg/kg/day, L-RTEE), medium (400 mg/kg/day, M-RTEE), or high (800 mg/kg/day, H-RTEE) doses of RTEE, and an LPS + Flu group receiving fluoxetine hydrochloride (10 mg/kg/day). Mice were kept under standardized conditions: an ambient temperature of 23 ± 2 °C, 40%–70% relative humidity, and a 12-h photoperiod, and had ad libitum access to food and water.

**FIGURE 1:**
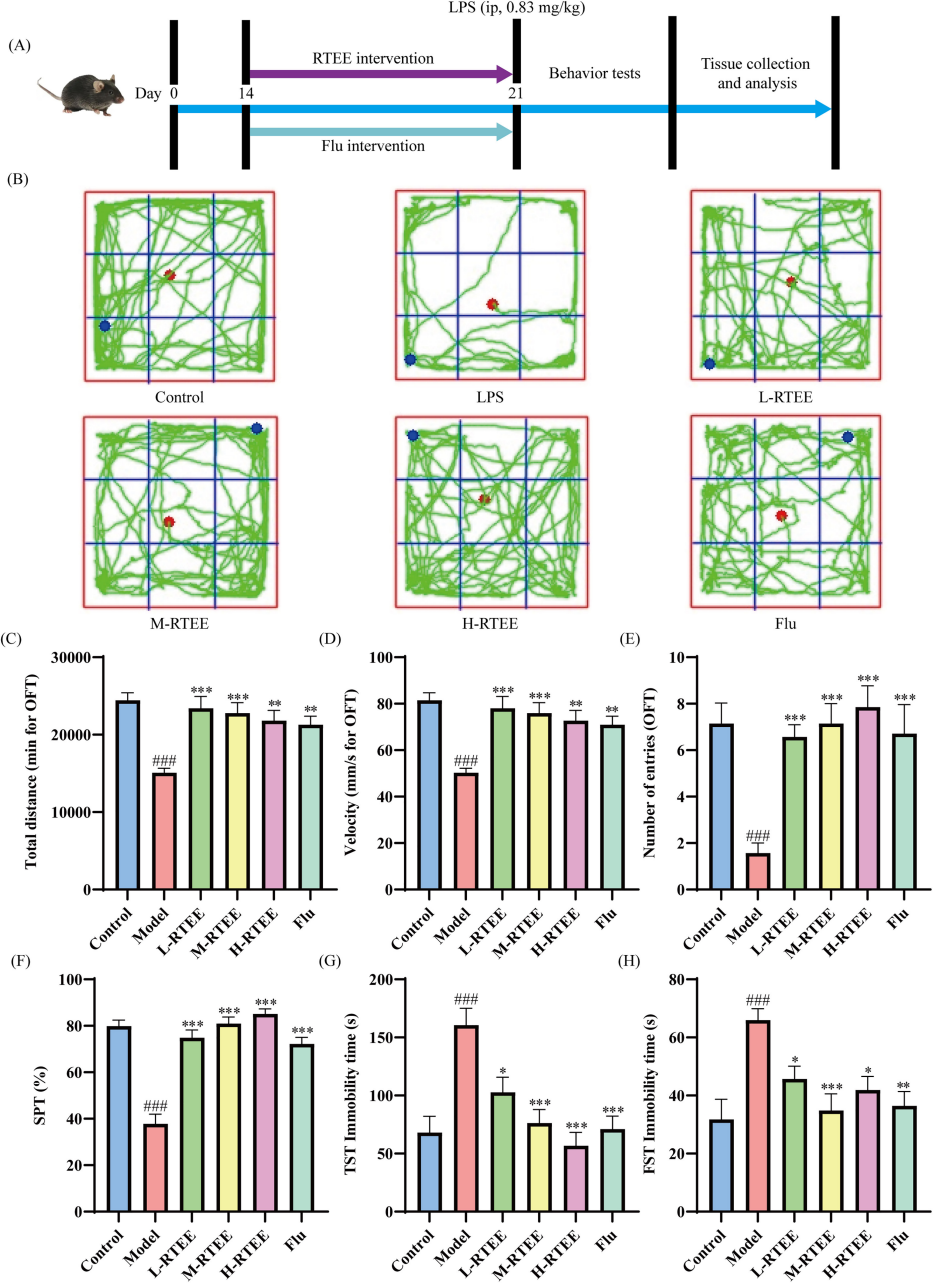
Effects of RTEE on depressive-like behaviors in LPS-challenged mice (*n* = 8). **(A)** Schematic of the experimental design. **(B)** Representative trajectories of mice in the OFT. **(C)** Total distance in the OFT. **(D)** Velocity in the OFT. **(E)** Center entries in the OFT. **(F)** Sucrose preference in the SPT. **(G)** Immobility time in the TST. **(H)** Immobility time in the FST. ^###^*p* < 0.001 vs control group; *p < 0.05, **p < 0.01 and ***p < 0.001 vs LPS group.

The LPS model was established following the protocol described by Wang et al. with minor modifications (15). The experimental design was presented in Figure 1A. Following adaptation, mice in the LPS + RTEE groups (L-, M-, and H-RTEE) and LPS + Flu group began to receive daily intervention for seven consecutive days. At day 8, mice in the treatment groups (L-, M-, H-RTEE, and Flu) and the LPS group were intraperitoneally injected with 0.83 mg/kg LPS (Sigma-Aldrich, #L2630, United States). Behavioral tests were conducted starting on day 9. Following behavioral assessments, mice were humanely sacrificed, and tissues were harvested for analysis.

### 2.3 Behavioral tests

#### 2.3.1 Sucrose preference test (SPT)

Sucrose preference test was employed to evaluate anhedonia in the animal model (16). Prior to testing, mice were deprived of food and water (12 h). Subsequently, two bottles (one with distilled water solution and the other with 1% sucrose) were provided *ad libitum* to the mice during the 48 h adaptation phase. Potential positional bias was minimized by alternating the bottle positions at 12-h intervals. Following adaptation, mice were deprived of food and water (24 h). For the test itself, mice were individually housed and given a choice between two bottles containing either distilled water or 1% sucrose solution. Following 4 h of ad libitum access to food and water, sucrose preference was quantified with the formula below:


$$\mathrm{Preference}\;\mathrm{for}\;\mathrm{sucrose}\left(\%\right)=\frac{\mathrm{Sucrose}\,\mathrm{water}\,\mathrm{consumption}}{\mathrm{Total}\,\mathrm{water}\,\mathrm{consumption}}\times 100\%$$

#### 2.3.2 Open field test (OFT)

Open field test was utilized to assess locomotor activity in the animal model in an unfamiliar setting (17). Each mouse was placed in the central area of a test chamber (50 × 50 × 50 cm) that was evenly divided into nine square areas, and the movement parameters (total distance, velocity, and center entries) over 5 min were automatically measured using the XR-VT101 VisuTrack software (Shanghai, China).

#### 2.3.3 Tail suspension test (TST)

Tail suspension test was utilized to evaluate behavioral despair in the animal model (18). Mice were lifted 30 cm above the TST module by securing a flexible rope loop to the distal 25% of the tail (measured from the base). The immobility time over 5 min was automatically measured using the the XR-VT101 VisuTrack software (Shanghai, China).

#### 2.3.4 Forced swim test (FST)

Forced swim test was also employed to assess behavioral despair in the animal model (19). Each mouse was placed in a cylindrical test chamber (Ø 10 × 30 cm) containing tap water (23 ± 2 °C) to a height of 22.5 cm. The immobility time over 5 min was automatically measured using the XR-VT101 VisuTrack software (Shanghai, China).

### 2.4 Immunofluorescence (IF)

Dissected brains were fixed in 4% paraformaldehyde (4 h), dehydrated in 30% sucrose, and finally embedded in optimal cutting temperature (OCT) compound. Coronal sections (20 μm) were cut on a Leica CM1950 cryostat (Wetzlar, Germany). Prior to immunostaining, the sections were rinsed with phosphate-buffered saline (PBS), permeabilized with 0.25% Triton X-100 in PBS (PBST) (30 min), and blocked with 10% horse serum solution (2 h). They were then probed with primary antibodies (14 h, 4 °C), purchased from Wako (anti-Iba-1 #019-19741; Tokyo, Japan) and Abcam (anti-NeuN #ab177487, anti-GFAP #ab7260; United Kingdom). Following PBST washes (1 h), the sections were probed with the secondary antibodies conjugated to fluorescein isothiocyanate (FITC) for 2 h at room temperature. Following further washes, the sections were counterstained with DAPI (Sigma-Aldrich, United States) and finally rinsed with PBS. Images were captured with a Leica DM2500 fluorescence microscope (Wetzlar, Germany), and Image J software was used to quantify the number of positive cells.

### 2.5 Cell culture and treatment

BV2 murine microglial cell line was obtained from the Shanghai Cell Bank (Shanghai, China). Following cultivation under standard conditions (37 °C, 5% CO_2_) using high-glucose DMEM containing 10% fetal bovine serum (FBS), cells were treated with RTEE (50, 100, and 200 μg/mL) in combination with or without 2 μg/mL LPS for 24 h.

### 2.6 Cell viability Assay

Cell viability of BV2 cells was evaluated using a Cell Counting Kit-8 (CCK8) assay kit (#36208ES, Yeasen, Shanghai, China). Following treatment, each well was treated with CCK-8 solution followed by incubation (25 min, 37 °C). Absorbance was then measured at 450 nm using a BioTek microplate reader (VT, United States).

### 2.7 Nitric oxide (NO) assay

The concentration of NO in the culture supernatant was measured using a NO content assay kit (#G0132W, Geruisi, Suzhou, China). In brief, nitrate in the culture supernatant was reduced to nitrite by nitrate reductase, and the resultant nitrite was then subjected to the Griess reaction, forming a colored azo compound. Absorbance was measured at 530 nm using a BioTek microplate reader (VT, United States).

### 2.8 Western blotting (WB)

Protein samples were prepared by homogenization in RIPA lysis buffer, separated by sodium dodecyl sulfate-polyacrylamide gel electrophoresis (SDS-PAGE) using 8%–10% gels, and electrotransferred onto methanol-activated polyvinylidene fluoride (PVDF) membranes (Millipore, MA, United States). Subsequently, the membranes were blocked with 5% skimmed milk (2 h). The membranes were probed with the primary antibodies listed in Table 1 (14 h, 4 °C). Following 0.1% Tween-20 in tris buffered saline solution (TBST) washes (1 h), the membranes were probed with the horseradish peroxidase (HRP)-linked secondary antibodies for 1 h at room temperature, sourced from Cell Signaling Technology (anti-mouse IgG #7076, anti-rabbit IgG #7074; MA, United States). Following further washes, protein bands were detected by enhanced chemiluminescence (ECL) using a commercial kit (#36208ES, Yeasen, Shanghai, China), and then imaged on a Tanon 5200 chemiluminescence imaging system (Shanghai, China). Band intensities were quantified by normalization to β-actin.

**TABLE 1:** List of primary antibodies for western blotting (WB).

Antibody	Source	Catalog number
Anti-TNF-α	Wanlei Bio (Shenyang, China)	WL01581
Anti-IL-6	Wanlei Bio (Shenyang, China)	WL02841
Anti-P-p65	Wanlei Bio (Shenyang, China)	WL02169
Anti-TLR4	Proteintech (IL, USA)	66350
Anti-MyD88	Proteintech (IL, USA)	67969
Anti-p65	Proteintech (IL, USA)	66535
Anti-PSD95	Cell Signaling Technology (MA, USA)	3450
Anti-P-JNK	Cell Signaling Technology (MA, USA)	4671
Anti-JNK	Cell Signaling Technology (MA, USA)	9252
Anti-P-p38	Cell Signaling Technology (MA, USA)	4511
Anti-p38	Cell Signaling Technology (MA, USA)	8690
Anti-P-IκBα	Cell Signaling Technology (MA, USA)	2859
Anti-IκBα	Cell Signaling Technology (MA, USA)	4812
Anti-NLRP3	ImmunoWay (TX, USA)	YM8024

### 2.9 Statistical analysis

All data were analyzed using GraphPad Prism software Version 9.5 (CA, United States) and are presented as means ± SEM. Group comparisons were conducted by one-way analysis of variance (ANOVA), with *p* < 0.05 deemed statistically significant.

## 3 Results

### 3.1 RTEE improved LPS induced depression-like behaviors in mice

Behavioral assessments, including SPT, OFT, TST, and FST were utilized to evaluate the effects of RTEE on LPS induced behavioral deficits in mice. The results showed that locomotor activity of LPS-challenged mice in the OFT was significantly decreased versus the control group, as evidenced by a significant reduction in total distance, velocity, and center entries (*p* < 0.001, Figures 1B–E). Moreover, sucrose preference in the SPT was significantly reduced in LPS-challenged mice (*p* < 0.001, Figure 1F), whereas the immobility time in both the TST and FST was significantly prolonged (*p* < 0.001, Figures 1G,H). However, RTEE treatment significantly reversed these behavioral abnormalities (*p* < 0.05, Figure 1). These data suggested that RTEE effectively improved LPS induced behavioral deficits in mice.

### 3.2 RTEE alleviated NeuN down-regulation in LPS-challenged mice

**FIGURE 2:**
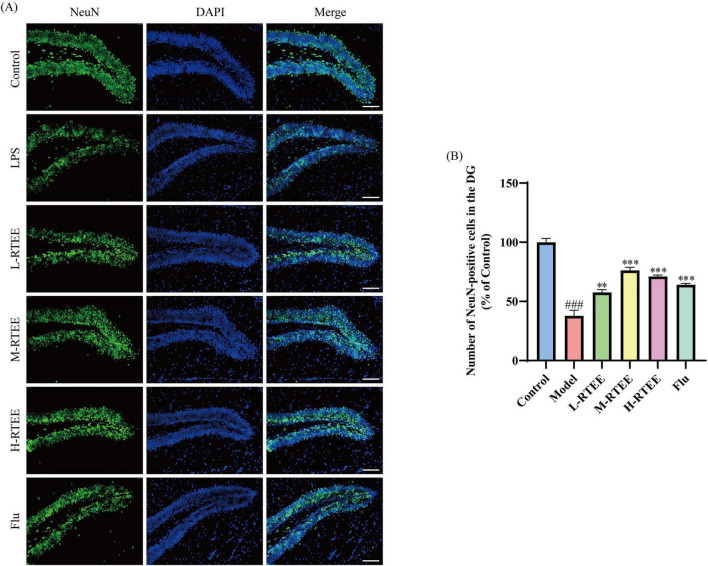
Effects of RTEE on NeuN^+^ neurons in the hippocampus of LPS-challenged mice (*n* = 3). **(A)** Representative IF staining images of NeuN^+^ neurons in the DG (scale bar: 100 μm). **(B)** Quantity analysis of NeuN^+^ cells. ^###^*p* < 0.001 vs control group; **p < 0.01 and ***p < 0.001 vs LPS group.

Lipopolysaccharide-induced neuroinflammation triggers significant neuronal loss (20). NeuN serves as a well-established marker for mature neurons in the CNS (21). The impact of RTEE on neuronal survival was assessed by NeuN IF staining. The results showed that LPS-challenged mice exhibited a marked decrease in NeuN^+^ cell numbers in the dentate gyrus (DG) compared with the control group (*p* < 0.001, Figure 2). Following RTEE intervention, NeuN^+^ cell numbers were significantly elevated (*p* < 0.01, Figure 2). This data suggested that RTEE potently alleviated NeuN down-regulation in the DG of LPS-challenged mice.

### 3.3 RTEE increased PSD95 expression in the hippocampus of LPS-challenged mice

**FIGURE 3:**
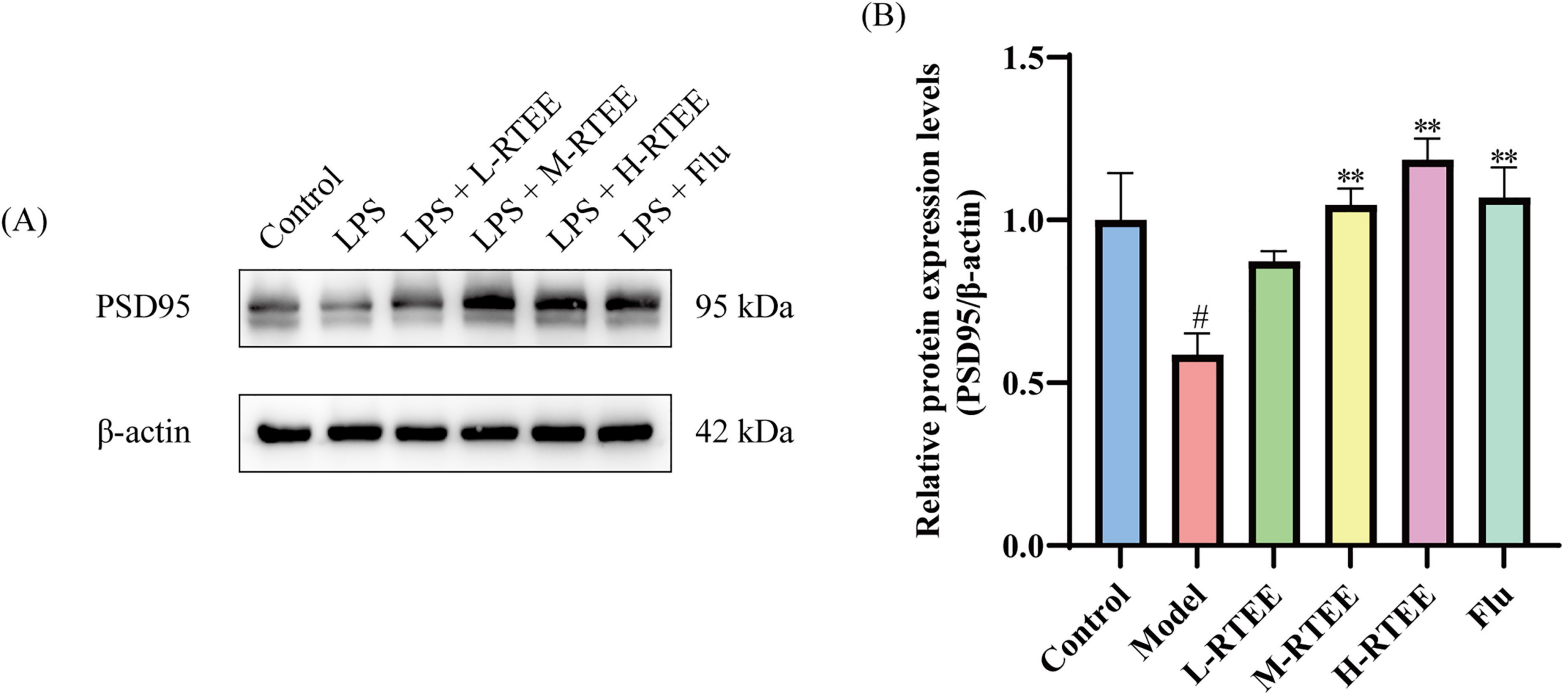
Effect of RTEE on the hippocampal PSD95 expression in LPS-challenged mice (*n* = 3). **(A)** Representative WB image of PSD95. **(B)** Quantitative analysis of PSD95. ^#^*p* < 0.05 vs control group; **p < 0.01 vs LPS group.

As a scaffold protein highly enriched in the postsynaptic density, PSD95 is essential for synaptic plasticity (22). WB was performed to assess the impact of RTEE on PSD95 expression. The results showed that LPS exposure significantly reduced the expression level of PSD95 in hippocampus compared with the control group (*p* < 0.05, Figure 3). However, RTEE treatment significantly up-regulated PSD95 expression (*p* < 0.01, Figure 3). This data suggested that RTEE potently counteracted the down-regulated PSD95 expression induced by LPS.

### 3.4 RTEE attenuated hippocampal neuroinflammation in LPS-challenged mice

**FIGURE 4:**
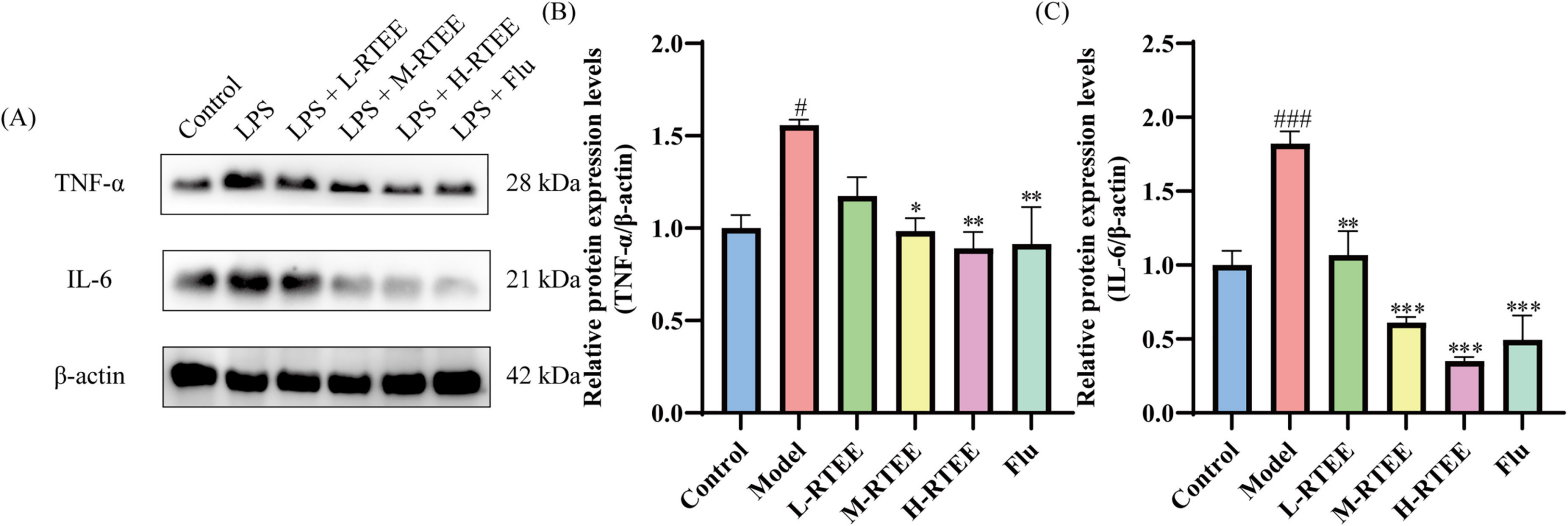
Effects of RTEE on the protein expression of inflammatory cytokines in the hippocampus of LPS-challenged mice (*n* = 3). **(A)** Representative WB images of TNF-α and IL-6. **(B–C)** Quantitative analysis of **(B)** TNF-α and **(C)** IL-6. ^#^*p* < 0.05 and ^###^*p* < 0.001 vs control group; *p < 0.05, **p < 0.01 and ***p < 0.001 vs LPS group.

Microglia and astrocytes, the essential glial cells in the central nervous system (CNS), undergo dynamic phenotypic transformations through reciprocal crosstalk, collectively regulating neuroinflammatory responses (23). LPS stimulation triggers rapid microglia activation, leading to elevated secretion of pro-inflammatory cytokines (24). WB was performed to assess the effects of RTEE on pro-inflammatory cytokine expression in the hippocampus. The results showed that LPS-challenged mice exhibited a significant increase in the expression levels of hippocampal pro-inflammatory cytokines (TNF-α and IL-6) compared with the control group (*p* < 0.05, Figure 4). However, RTEE treatment significantly down-regulated the levels of these elevated levels (*p* < 0.05, Figure 4). These findings suggested that RTEE potently reduced the levels of pro-inflammatory cytokines.

**FIGURE 5:**
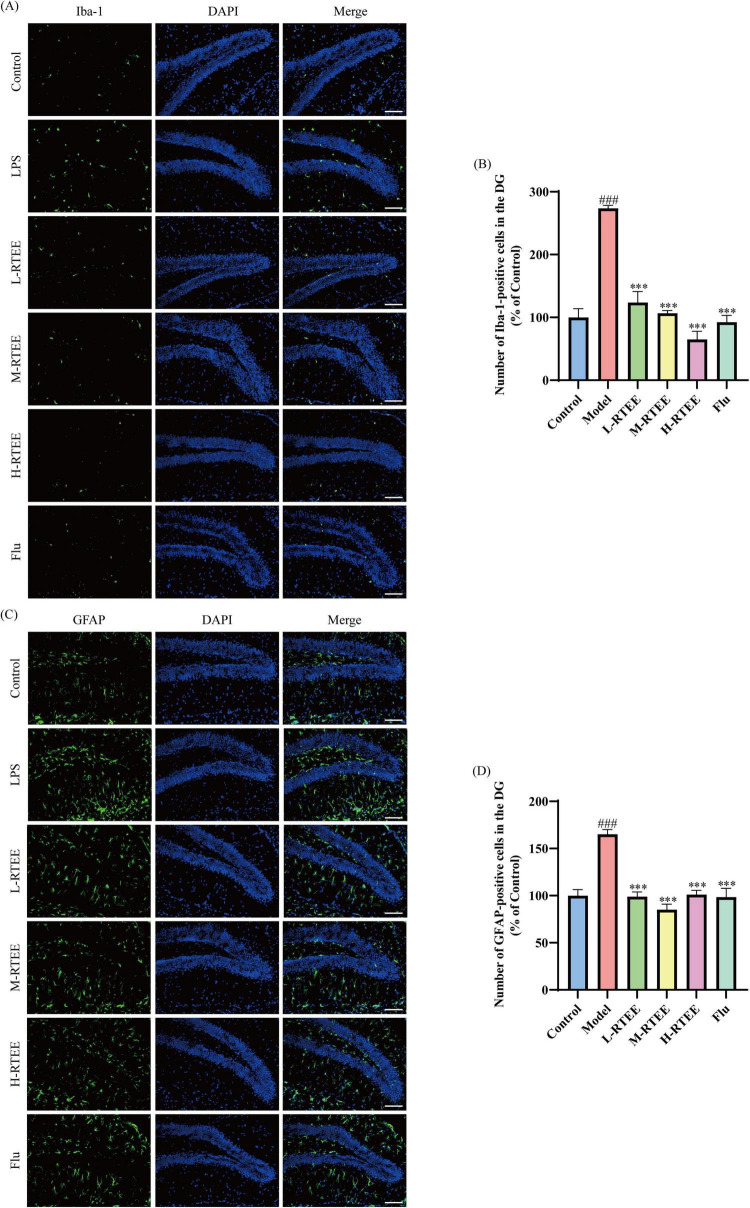
Effects of RTEE on hippocampal neuroinflammation in LPS-challenged mice (*n* = 3). **(A)** Representative IF staining images of Iba-1^+^ microglia in the DG (scale bar: 100 μm). **(B)** Quantity analysis of Iba-1^+^ cells. **(C)** Representative IF staining images of GFAP^+^ astrocytes in the DG (scale bar: 100 μm). **(D)** Quantitative analysis of GFAP^+^ cells. ^###^*p* < 0.001 vs control group; ***p < 0.001 vs LPS group.

Iba-1 and GFAP, the classic markers for activated microglia and reactive astrocytes, jointly characterize neuroinflammatory responses (25, 26). The impact of RTEE on neuroinflammation was assessed by Iba-1 and GFAP IF staining. The results showed that LPS-challenge mice exhibited a significant reduction in Iba-1^+^ and GFAP^+^ cell numbers in the DG versus the control group (*p* < 0.001, Figure 5). Following RTEE intervention, the number of Iba-1^+^ and GFAP^+^ cells were markedly reduced (*p* < 0.001, Figure 5). These data suggested that RTEE effectively attenuated neuroinflammation.

### 3.5 RTEE attenuated neuroinflammation via inhibiting the TLR4/MyD88/MAPK/ NF-κB/NLRP3 signaling pathway in the hippocampus of LPS-challenged mice

**FIGURE 6:**
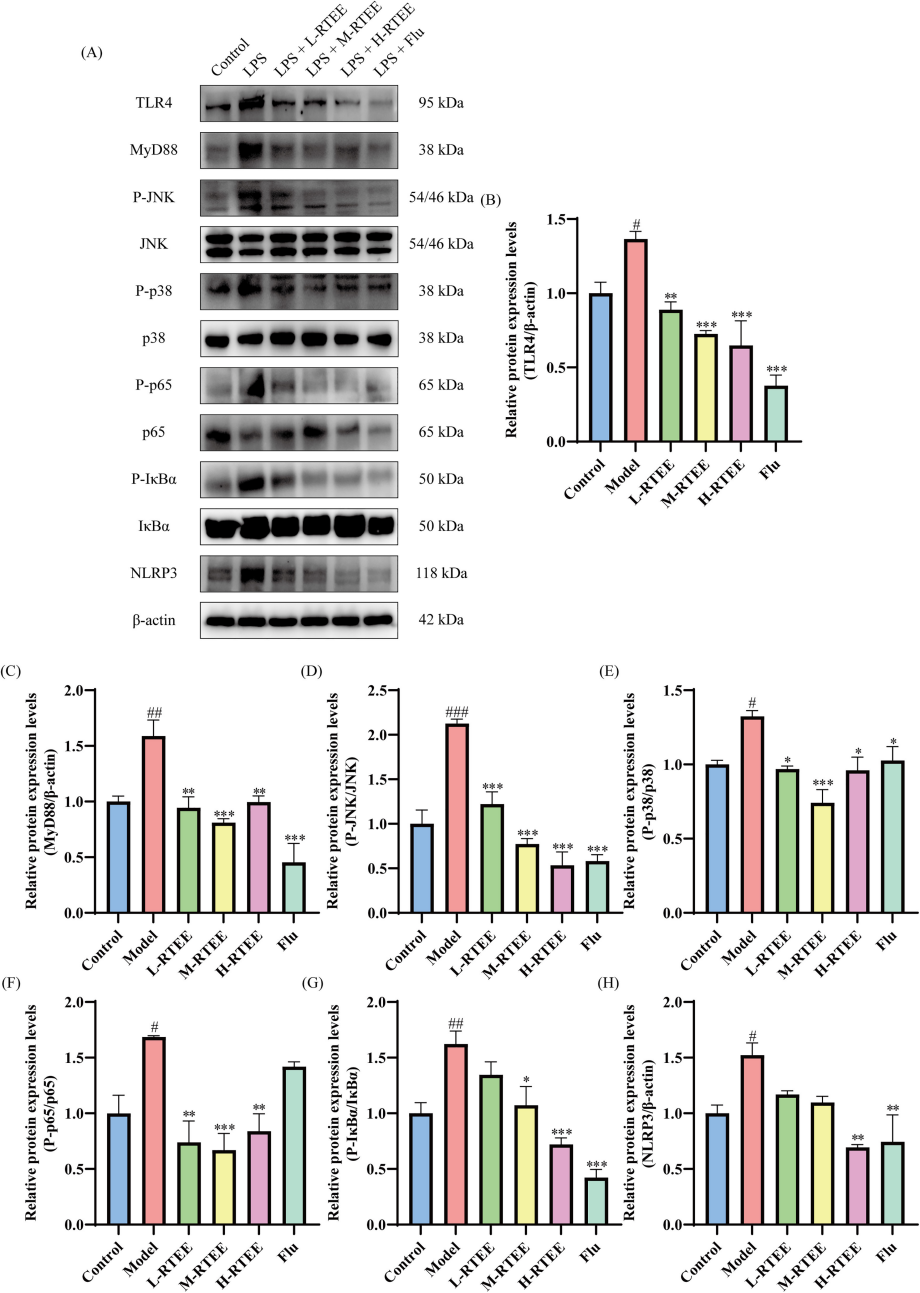
Effects of RTEE on the hippocampal TLR4/MyD88/MAPK/NF-κB/NLRP3 signaling pathway in LPS-challenged mice (*n* = 3). **(A)** Representative WB images of TLR4, MyD88, P-JNK, JNK, P-p38, p38, P-p65, p65, P-IκBα, IκBα, and NLRP3. **(B–H)** Quantitative analysis of **(B)** TLR4, **(C)** MyD88, **(D)** the ratio of P-JNK/JNK, **(E)** the ratio of P-p38/p38, **(F)** the ratio of P-p65/p65, **(G)** the ratio of P-IκBα/IκBα, and **(H)** NLRP3. ^#^*p* < 0.05, ^##^*p* < 0.01 and ^###^*p* < 0.001 vs control group; *p < 0.05, **p < 0.01 and ***p < 0.001 vs LPS group.

The TLR4/MyD88/MAPK/NF-κB/NLRP3 signaling pathway represents a core mechanism underlying the regulation of inflammatory responses. WB was performed to assess the effects of RTEE on the TLR4/MyD88/MAPK/NF-κB/NLRP3 signaling pathway in the hippocampus. The results showed that the expression level of TLR4, MyD88, and NLRP3, as well as the P-JNK/JNK, P-p38/p38, P-p65/p65, and P-IκBα/IκBα ratios, were significantly1 elevated in the hippocampus of LPS-challenged mice compared with the control group (*p* < 0.05, Figure 6). However, RTEE treatment significantly decreased the levels of these inflammatory signaling molecules (*p* < 0.05, Figure 6).

### 3.6 RTEE rescued cell viability and reduced NO production in LPS-challenged BV2 cells via suppressing the NF-κB signaling pathway

**FIGURE 7:**
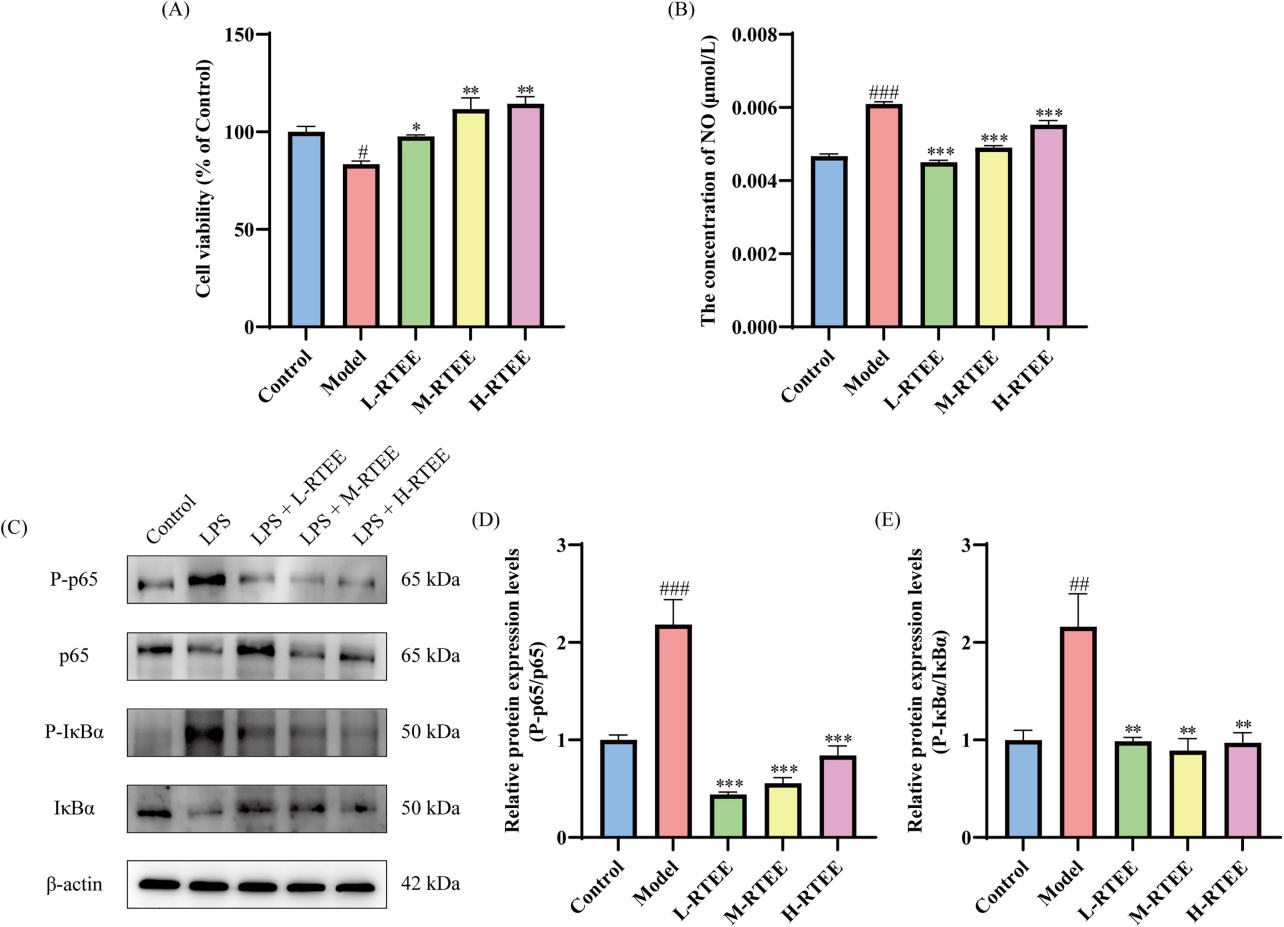
Effects of RTEE on LPS-challenged BV2 cells. (*n* = 3). **(A)** Cell viability. **(B)** The concentration of NO in the culture supernatant. **(C)** Representative WB images of P-p65, p65, P-IκBα, and IκBα. **(D–E)** Quantitative analysis of **(D)** the ratio of P-p65/p65 and **(E)** the ratio of P-IκBα/IκBα. ^#^*p* < 0.05, ^##^*p* < 0.01 and ^###^*p* < 0.001 vs control group; *p < 0.05, **p < 0.01 and ***p < 0.001 vs LPS group.

High-dose LPS treatment triggered inflammatory responses and apoptosis in BV2 cells, resulting in a significant reduction in cell viability and a marked increase in NO production (27). The results demonstrated that LPS-exposed BV2 cells showed a significant reduction in cell viability (*p* < 0.05, Figure 7A), along with a marked increase in the concentration of NO in the culture supernatant compared with the control group (*p* < 0.001, Figure 7B). Following RTEE intervention, these abnormalities were significantly reversed (*p* < 0.05, Figure 7). These data suggested that RTEE potently rescued cell viability and reduced NO production in BV2 cells challenged with LPS.

Lipopolysaccharide exposure activates the NF-κB signaling axis in microglia (28). WB was performed to assess the impact of RTEE on the NF-κB signaling pathway in LPS-challenged BV2 cells. The results showed that the ratios of P-p65/p65 and P-IκBα/IκBα were significantly increased in LPS-exposed BV2 cells versus the control group (*p* < 0.01, Figures 7C–E). Following RTEE intervention, these inflammatory signaling molecules were significantly reduced (*p* < 0.01, Figures 7C–E). These findings suggested that RTEE potently inhibited the NF-κB signaling pathway in LPS-challenged BV2 cells.

## 4 Discussion

*R. tomentosa* fruit, a dietary food, is traditionally used for its aphrodisiac and antidiarrheal properties. Nevertheless, its antidepressant effects remain to be thoroughly investigated. In this study, RTEE was prepared for intervention. Given that the chemical composition of *R. tomentosa* fruit is highly dependent on its degree of maturity, fully ripe fruit should be used for RTEE preparation, with strict adherence to the procedure described herein to ensure reproducibility (14, 29). For the *in vivo* experiments, depression-like behaviors were reliably induced by LPS, as confirmed by behavioral tests. Notably, RTEE effectively improved depression-like behaviors in mice, exhibiting significant antidepressant effects.

Depression-like behaviors are strongly associated with neuronal loss, impaired synaptic plasticity (30). Due to the limited proliferative capacity of adult neurons, excessive neuronal loss can impair normal brain function, a deficit potentially resulting from LPS induced apoptosis (31, 32). In addition, neuroinflammation triggered by LPS leads to marked cognitive dysfunction in mice (33). The downregulation of NeuN, a marker of post-mitotic neurons, may be closely associated with impaired brain function and stress responses (34). Liu et al. found a significant decrease in NeuN^+^ cell numbers in the DG of LPS-challenged mice (35). In addition, Li et al. observed a significant downregulation of PSD95 expression in the hippocampus of mice expose to LPS (36). Our data confirmed these observations. By contrast, RTEE significantly increased NeuN^+^ cell numbers and enhanced PSD95 expression in the hippocampus, thereby alleviating neuronal loss and restoring synaptic plasticity.

Neuroinflammation considerably contributes to the occurrence and development of depression, while depression further exacerbates inflammatory responses that aggravates disease (37). Microglia, the CNS-resident immune cells, can polarize into either the pro-inflammatory M1 phenotype (releasing neurotoxic mediators) or the anti-inflammatory M2 phenotype (secreting neuroprotective factors). Under sustained neuroinflammatiory stimulation, the homeostasis between M1 and M2 polarization is disrupted, leading to a predominant shift toward the neurotoxic M1 phenotype (38). Astrocytes similarly exhibit phenotypic plasticity, dynamically polarizing between neurotoxic (A1) and neuroprotective (A2) states. Notably, activated microglia promotes A1 astrocytic polarization, consequently amplifying inflammatory responses (39). Iba-1 and GFAP are classic molecular indicators for evaluating neuroinflammation, reflecting the activation of microglia and astrocytes, respectively. Ali et al. observed a significant increase in Iba-1^+^ and GFAP^+^ cell numbers in the DG of LPS-challenged mice (40). Our results corroborated these observations. In contrast, RTEE markedly decreased Iba-1^+^ and GFAP^+^ cell numbers, thus attenuating neuroinflammation.

TLR4 serves as the major pattern recognition receptor for LPS. Following LPS binding, TLR4 recruits MyD88, triggering downstream signaling cascades, including the MAPK and NF-κB signaling pathways. Subsequently, this MyD88-dependent pathway promotes the assembly of NLRP3 (41). NLRP3 acts as a key initiator of pyroptosis, an inflammatory programmed cell death, thereby contributing to various neurological diseases (42). Previous study demonstrated that the hippocampal TLR4/MyD88/MAPK/NF-κB/NLRP3 signaling pathway was significantly activated in LPS-challenged mice, with elevated levels of TNF-α and IL-6 (43–45). Our findings are consistent with these results. Conversely, RTEE attenuated hippocampal inflammation by inhibiting the TLR4/MyD88/MAPK/NF-κB/NLRP3 signaling pathway to reduce the levels of pro-inflammatory cytokines. Furthermore, *in vitr*o results showed that RTEE significantly inhibited the NF-κB signaling pathway in LPS-challenged BV2 cells, further validating its anti-neuroinflammatory effect.

As a selective serotonin reuptake inhibitor (SSRI), fluoxetine represents a first-line pharmacological option in the clinical management of depression (46). However, SSRIs are well-documented to cause sexual dysfunction as a common adverse effect. Although fluoxetine has a relatively favorable adverse effect profile among SSRIs, it still may impair sperm function (47). Additionally, emerging evidence suggests that fluoxetine may induce unexpected adverse effects such as alopecia, systemic hypertension and urinary retention (48–50). Against this backdrop, this study focused on RTEE and demonstrated its antidepressant effects in an LPS induced depression model, thereby suggesting its potential as a complementary or alternative therapeutic strategy to conventional treatments.

As proteins are fundamental to biological functions, most cellular functions rely on specific protein-protein interactions, such as those involved in signaling pathways (51). Previous study demonstrated that mRNA and protein expression level are often poorly correlated, and that the biological functions attributed to differential mRNA expression frequently depend on corresponding changes at the protein level (52). Therefore, this study investigated the molecular mechanism by which RTEE ameliorated LPS-induced depression-like behaviors in mice at the protein level, using combined *in vivo* and *in vitro* methods. Investigation at the mRNA level was beyond the scope of this study. Notably, given that LPS induced depression model represents an acute inflammatory challenge rather than a chronic model of depression, the long-term effects of RTEE warrant further investigation. Sălcudean et al. revealed that neuroinflammation can activate the HPA axis and impairs serotonin synthesis, suggesting that LPS induced neuroinflammation may disrupt the HPA axis and monoaminergic neurotransmitter systems, potentially collectively contributing to depression-like behaviors in mice (37). Although this study did not assess the effects of RTEE on the HPA axis or monoaminergic systems, the finding that RTEE attenuated neuroinflammation, associated with inhibition of the TLR4/MyD88/MAPK/NF-κB/NLRP3 signaling pathway, raised the possibility that its antidepressant effects may extend beyond neuroinflammation to engage the HPA axis and monoaminergic systems. Thus, future studies including the HPA axis, monoaminergic systems, and functional assays could lead to a more comprehensive mechanistic understanding of the antidepressant effects of RTEE.

**FIGURE 8:**
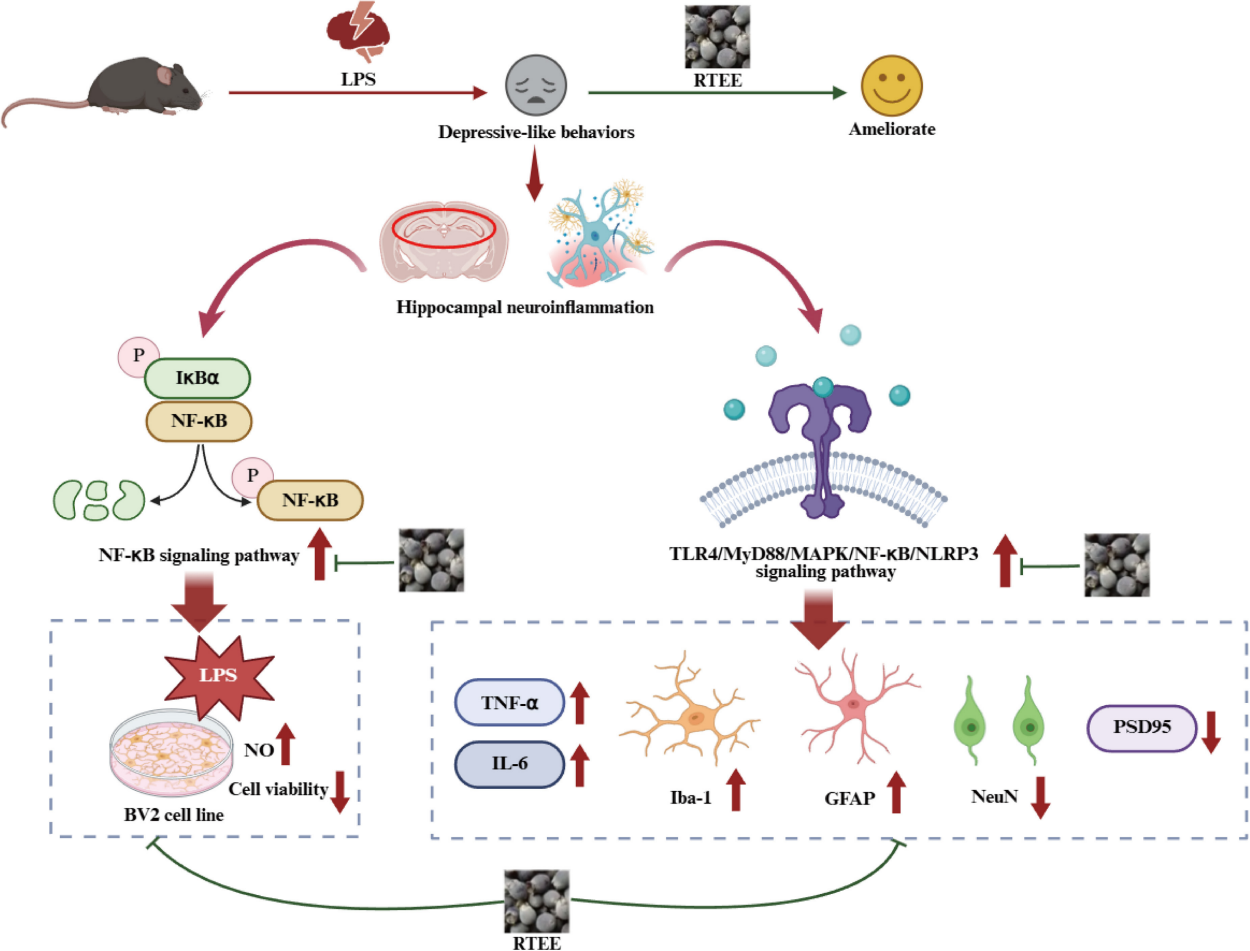
Mechanisms of RTEE ameliorating LPS induced depressive-like behaviors.

In conclusion, RTEE improves LPS induced depression-like behaviors in mice by attenuating neuroinflammation (Figure 8). These data suggest that *R. tomentosa* fruit may serve as a potential nutritional strategy for depression, with its anti-neuroinflammatory effects representing one of the contributing mechanisms.

## Data Availability

The original contributions presented in this study are included in this article/supplementary materials, further inquiries can be directed to the corresponding authors.
